# MicroRNA-34a Enhances T Cell Activation by Targeting Diacylglycerol Kinase ζ

**DOI:** 10.1371/journal.pone.0077983

**Published:** 2013-10-17

**Authors:** Jinwook Shin, Danli Xie, Xiao-Ping Zhong

**Affiliations:** 1 Department of Pediatrics, Division of Allergy and Immunology, Duke University Medical Center, Durham, North Carolina, United States of America; 2 School of Laboratory Medicine, Wenzhou Medical University, Wenzhou, Zhejiang, China; 3 Department of Immunology, Duke University Medical Center, Durham, North Carolina, United States of America; University of Iowa, United States of America

## Abstract

The engagement of the T cell receptor (TCR) induces the generation of diacylglycerol (DAG), an important second messenger activating both the Ras/Erk and PKCθ/NFκB pathways. DAG kinases (DGKs) participate in the metabolism of DAG by converting it to phosphatidic acid. DGKζ has been demonstrated to be able to inhibit DAG signaling following TCR engagement. Deficiency of DGKζ increases the sensitivity of T cells to TCR stimulation, resulting in enhanced T cell activation ex vivo and in vivo. However, the mechanisms that control DGKζ expression are poorly understood. Here we demonstrate that DGKζ mRNA is a direct target of a cellular microRNA miR-34a. The DGKζ transcript is decreased, whereas the primary miR-34a is upregulated upon TCR stimulation. Ectopic miR-34a expression suppresses DGKζ protein expression through the seed match binding to both the 3' untranslated region and coding region of DGKζ mRNA, leading to increased ERK1/2 phosphorylation and surface expression of the T cell activation marker CD69 following TCR cross-linking. In contrast, overexpression of a miR-34a competitive inhibitor increases DGKζ expression and suppresses TCR-mediated T cell activation. Together, our data demonstrate that miR-34a is a negative regulator for DGKζ and may play an important role in regulating T cell activation.

## Introduction

T cell receptor (TCR) signaling is important for appropriate T cell development in the thymus and for function in the periphery. Following the engagement of TCR, the proximal tyrosine kinases phosphorylate adaptor molecules LAT and SLP76 lead PLCγ1 recruitment and formation of a multimolecular signal complex [1,2]. Activated PLCγ1 produces inositol 1,4,5-trisphosphate (IP_3_) and diacylglycerol (DAG) by hydrolyzing phosphatidylinositol 4,5-bisphosphate. IP_3_ triggers cellular calcium release, leading to NFAT nuclear translocation [3]. DAG activates both the RasGRP1/Ras/Erk and protein kinase C θ (PKCθ)/NFκB pathways. DAG kinases (DGKs) phosphorylate DAG to produce phosphatidic acid (PA), resulting in attenuation of DAG signaling [4-9]. Overexpression of DGKζ inhibits TCR-mediated Erk1/2 phosphorylation and upregulation of the T cell activation marker CD69 in Jurkat T cells [10]. DGKζ-deficient T cells show enhanced proliferation and the CD69 expression following TCR stimulation in vitro. In vivo, DGKζ differentially controls primary and memory antiviral immune responses following lymphocytic choriomeningitis virus infection [5,11,12]. Moreover, DGKζ deficiency prevents the induction of T cell anergy and promotes antitumor immunity [5,13]. Together with DGKα, DGKζ also promotes T cell maturation during intrathymic development in part by generating PA. Although the importance of DGKζ in the immune system has become clear [14], the mechanism by which DGKζ expression is regulated is poorly understood. 

 MicroRNAs (miRNAs), non-coding RNAs of 22−24 nucleotides in length, downregulate gene expression by binding to target messenger RNAs. They play important roles in various biological processes [15]. Recently it has been discovered that miRNAs contribute to the immune system as a new regulator of immune cell development and function [16]. For example, in T cells, aberrant function of Dicer, which is essential for miRNA biosynthesis, causes compromised αβ T cell lineage development and abnormal T helper cell proliferation and survival [17,18]. Many miRNAs have been demonstrated to play important roles in T cells. For example, miR-155 inhibits IFN-γ signaling by targeting the 3' untranslated region (UTR) of IFN-γRα but promotes Th1 differentiation through repressing c-Maf in CD4^+^ T cells [19-21]. It also targets suppressor of cytokine signaling 1 (SOCS1) to maintain competitive fitness of Foxp3^+^ regulatory T cells [22]. miR-181a downregulates multiple phosphatases to modulate TCR signaling during T cell development [23]. In the present study, we investigate whether DGKζ can be controlled by miRNAs. We demonstrate that miR-34a is upregulated following TCR stimulation. Moreover, miR-34a suppresses the expression of DGKζ, suggesting that miR-34a may function as an inhibitor of DGKζ to enhance T cell activation.

## Methods

### Mice and cell culture

C57BL/6 mice were purchased from Jackson laboratory. All mice were housed in a pathogen-free facility. This study was carried out in strict accordance with the recommendations in the *Guide for the Care and Use of Laboratory Animals of the National Institutes of Health*. All mice were used according to protocols approved by the Institutional Animal Care and Use Committee of Duke University (Protocol Number: A132-10-5). Cells from the spleen and lymph node were cultured in IMDM (Sigma-Aldrich) supplemented with 10% FBS (Hyclone), 100 U/mL penicillin G, 100 U/mL streptomycin, and 50μM β-mercaptoethanol at 37° C and 5% CO_2_ incubator. HEK293T and Jurkat T cells were grown in complete DMEM and RPMI-1640 media, respectively. 

### Generation of stable cell lines

Jurkat T cells were transduced with lentivirus expressing GFP alone or GFP plus pri-miR-34a or sponge miR-34a (SPN-34), and then the GFP^+^ transduced cells were sorted by flow cytometry (FACS). The lentiviral constructs expressing pri-miR-34a or SPN-34 have been previously described [24].

### T cell stimulation and flow cytometry

To activate T cells, splenocytes and lymph node cells from mice were incubated with an anti-CD3ε antibody (clone 145-2C11, 1 ug/ml) and anti-CD28 (clone 37.51, 0.5 ug/ml) antibodies for 48 hours. After staining the cells with fluorochrome-conjugated anti-CD4 and anti-CD8 antibodies, the labeled cells were sorted by FACS. Sorted CD44^lo^CD62L^+^ naïve T cells were also used for the experiment. Stable Jurkat T cells were incubated with Jurkat T cell–specific anti-TCR ascites (clone C305, 1:50000), and the CD69 surface expression was then measured by FACS (BD FACSCanto II). To determine Erk1/2 phosphorylation, cells were fixed with 1.6% paraformaldehyde, permeablized with 100% methanol, and stained with anti-phospho-Erk1/2 (Cell Signaling Technology) and anti-rabbit PE secondary antibodies. The collected data were analyzed using FlowJo Version 9.2 software (Tree Star). 

### Quantitative real-time PCR

Total RNAs were extracted from sorted T cells using TRI reagent (Sigma), and cDNAs were prepared using an iScrip cDNA Synthesis Kit (Bio-Rad). DGKζ mRNA and primary miR-34a (pri-miR-34a) expressions were measured using SsoFast EvaGreen Supermix (Bio-Rad). The used primer pairs were as follows: mDGKζ, 5'-CTGAGGAGCAGATCCAGAGC-3' and 5'-TCCCCGACATAGCAGAAGTC-3'; pri-miR-34a, 5'-TTGGCAGTGTCTTAGCTGGTT-3' and 5'- TTGCTGACCTCTGACCTTTTC-3'; hDGKα, 5’-CCAAGGAGAGGGGCCTAATA-3’ and 5’-GCCATCCTCGAAGAGCTTTA-3’; hIL-2, 5’-TACAACTGGAGCATTTACTG-3’ and 5’-GTTTCAGATCCCTTTAGTTC-3’; hGAPDH, 5’-GAGTCCACTGGCGTCTTCA-3’ and 5’-GGGGTGCTAAGCAGTTGG-3’.

### Western blot

293T or Jurkat T cells were lysed with 1% Triton X-100 in PBS containing a protease inhibitor cocktail, and equal amounts of lysates were used for a western blot. Used antibodies are as follows: anti-mouse DGKζ [11], anti-human DGKζ (Abgent), anti-flag (Sigma), anti-p38, and anti-β-actin (Santa Cruz Biotechnology). A flag-DGKζ-MM bearing a mutated seed match (CACTGCC to CACCGCA ) was generated from flag-DGKζ-WT as a template with 5'-GCTTCCTGGACGCCACCACTGCCAGCCGCTTCTACAGGATCG-3' and its reverse complement primers using a QuikChange Site-Directed Mutagenesis Kit (Stratagene).

### Luciferase reporter assay

A reporter assay using luciferase was performed as previously described [25]. 293T cells in a 24-well dish were cotransfected with internal control renilla luciferase (Rluc), reporter firefly luciferase (Fluc) with or without DGKζ 3' UTR, and either empty or miR-34a expressing plasmid using FuGENE6 (Roche). Fluc and Rluc activities were assayed using the Dual Luciferase Reporter Assay System (Promega) 48 hours after transfection, and Fluc activity was then normalized by Rluc activity. Fluc-DGKζ 3' UTR construct was generated from 293T cell cDNAs using 5'-GATCCTCGAGCGGGCCGCCCACGGGCAGCAGG-3' and 5'-GATCGCGGCCGCGCACAGTCCGCGATGAAATGAC-3' primers.

### Statistical analysis

Statistical significance was calculated using a two-tail Student’s *t* test. The *p* values are defined as follows: *P < 0.05, **P < 0.01, ***P < 0.001. 

## Results

### Inversed expression pattern of DGKζ and miR-34a in naïve and activated T cells

**Figure 1:**
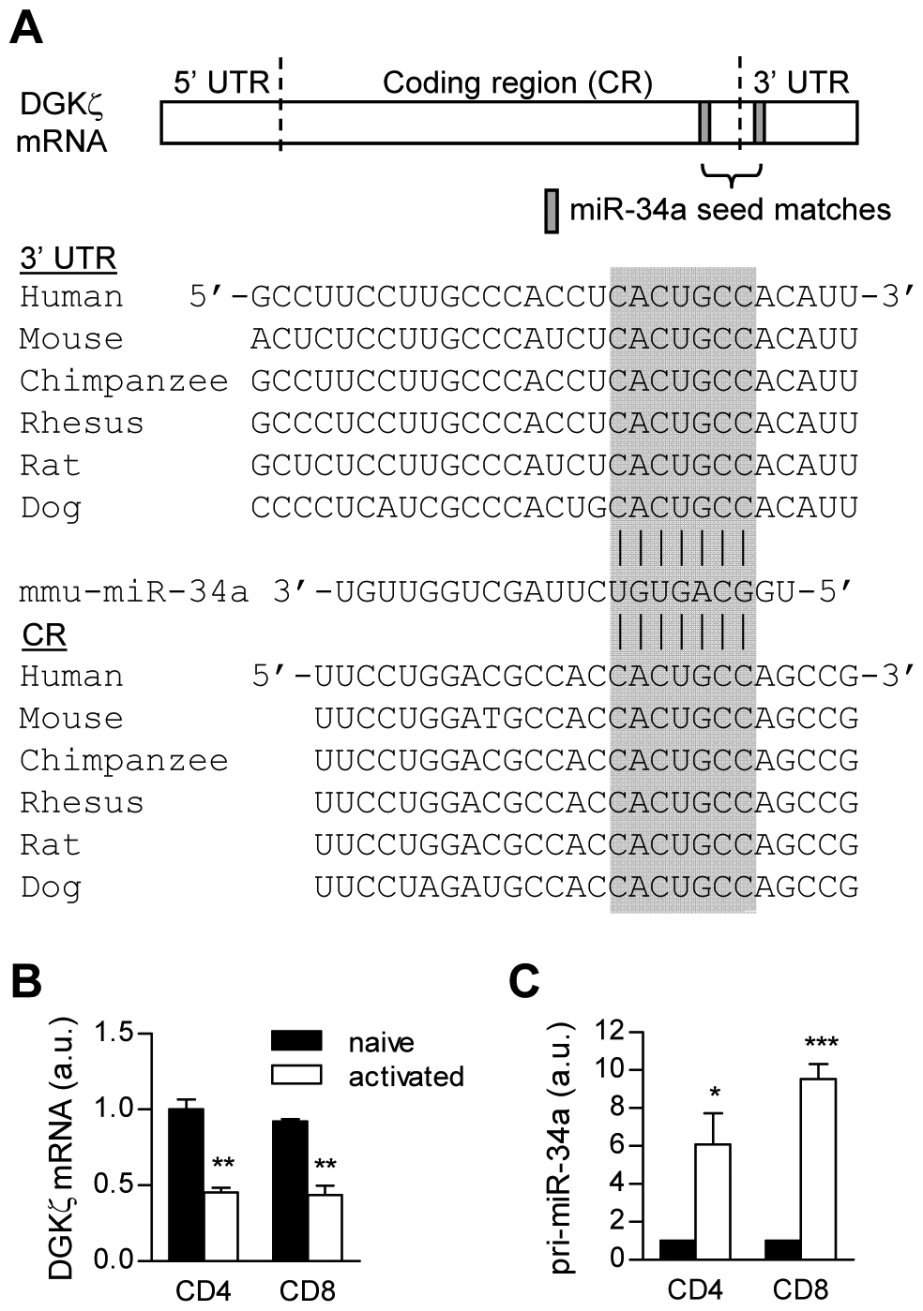
miR-34a and DGKζ mRNA expression in naïve and effector T cells. (**A**) Conserved seed matches on DGKζ mRNA are designated with the gray box. (**B** and **C**) Naïve T cells sorted from wild-type mice were incubated with anti-CD3ε and anti-CD28 antibodies for 48 hours. mRNAs from naïve and activated CD4^+^ and CD8^+^ T cells were reversely transcribed. miR-34a and DGKζ levels were quantified by real-time qPCR. Bar graphs represent mean ± SEM from three independent experiments (a.u., arbitrary unit; ***P < 0.001).

 Using a target prediction program, miRecords [26], we identified two conserved seed match sequences of miR-34a within both the 3' untranslated region (3' UTR) and the coding region (CR) of DGKζ (Figure 1). Given the important roles of DGKζ in T cells, we investigated the expressed patterns of DGKζ and miR-34a transcript during T cell activation. Periphery naïve CD62L^hi^CD44^low^ CD4^+^ and CD8^+^ T cells were stimulated by anti-CD3ε and anti-CD28 antibodies for forty-eight hours. Both DGKζ mRNA and miR-34a from sorted cells were quantified by real-time quantitative RT-PCR (qPCR) following reverse transcription. As shown in Figures 1B and 1C, DGKζ mRNA was downregulated in both activated CD4^+^ and CD8^+^ T cells, whereas the amount of miR-34a transcript was increased about six- to ten-fold, in comparison with CD44^low^ naïve T cells (Figures 1B and 1C). In contrast to miR-34a, we could not detect miR-34b/c in these cells (data not shown). These data reveal differential expression of miR-34a and DGKζ during T cell activation.

### DGKζ Is a Direct Target of miR-34a

**Figure 2:**
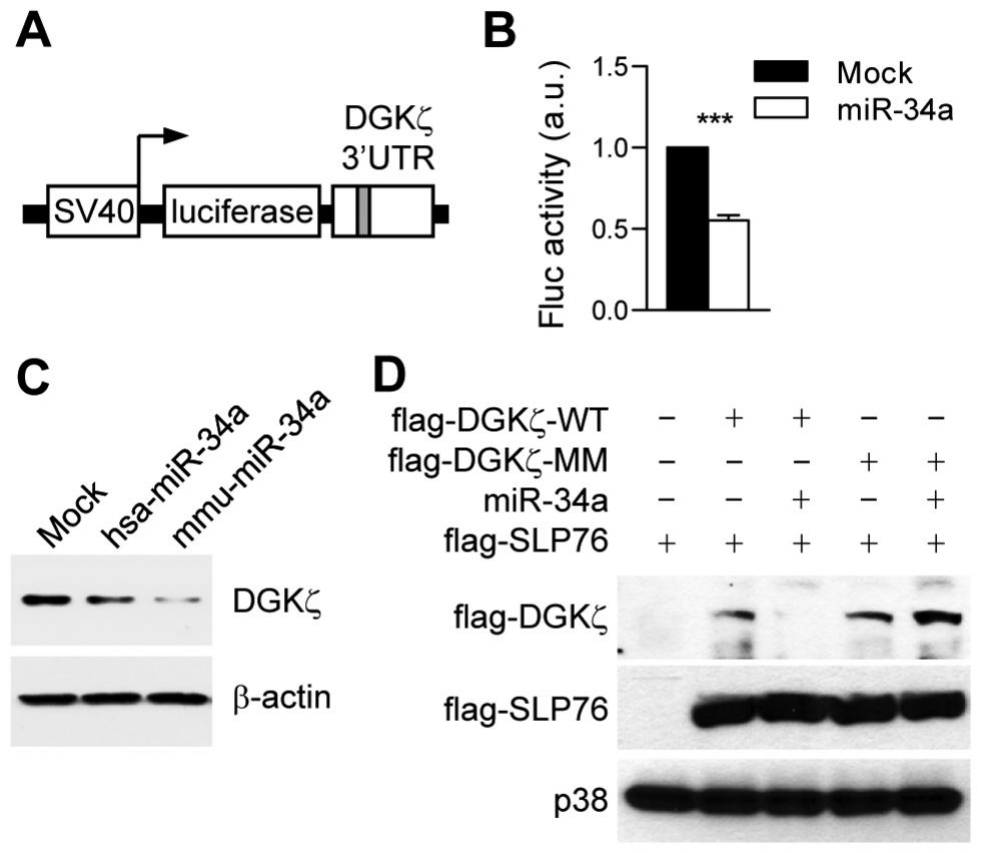
miR-34a directly represses DGKζ expression through targeting both DGKζ 3' UTR and CR. (**A**) Schematic reporter representation of construct to test the function of DGKζ 3' UTR. SV40, SV40 promoter. (**B**) Fluc reporter bearing DGKζ 3' UTR was cotransfected into 293T cells with Rluc reporter control and either miR-34a or empty plasmid (mock). Twenty-four hours later, luciferase activities were measured by a luminometer, and Fluc results were normalized by Rluc activities. Bar graphs represent mean ± SEM from three independent experiments (a.u., arbitrary unit; ***P < 0.001). (**C**) 293T cells were stably transfected with plasmid expressing human (hsa)- or mouse (mmu)-originated miR-34a, and a western blot was carried out for endogenous DGKζ protein. β-actin was used as a loading control. (**D**) 293T cells were transiently transfected with flag-tagged wild-type DGKζ (flag-DGKζ-WT), flag-DGKζ-MM bearing mutated seed match (CACTGCC to CACCGCA ), a cotransfected flag-SLP76 as an internal control, and miR-34a plasmids as indicated combinations. Twenty-four hours after transfection, the cells were harvested, and a western blot was conducted using an anti-flag antibody. p38 was used as a loading control for total lysates.

 The 5' seed sequence positioned in 2−8 neucleotides of miRNA is a critical region for target recognition and base pairing [27]. To confirm whether miR-34a can regulate DGKζ expression via their seed matches, we first generated a reporter construct containing Fluc open reading frame followed by 3' UTR from DGKζ (Figure 2A). This Fluc reporter was cotransfected with a vector expressing the Rluc for control of transfection efficiency into 293T cells with or without a plasmid expressing miR-34a. Fluc activity was measured, and then normalized using Rluc activity. The Fluc reporter activity decreased when miR-34a was cotransfected (Figure 2B). Moreover, overexpressed miR-34a can repress the endogenous DGKζ protein expression in 293T cells (Figure 2C). These data demonstrate that miR-34a can act directly on DGKζ 3' UTR (Figure 2B). The target sites of plant miRNAs are predominantly located on CR, but animal miRNA target studies have been mainly limited in UTRs [28]. Recent studies have validated several functional miRNA target sites in CRs in mammals [29-31]. Since the CR of DGKζ also contains a putative target site for miR-34a (Figure 1A), we next examined whether miR-34a could affect the expression of DGKζ protein by targeting the DGKζ CR via this target site. We generated plasmids containing coding sequences for DGKζ with the CR seed sequence intact (DGKζ-WT) or mutated (DGKζ-MM). To distinguish the exogenous DGKζ from the endogenous DGKζ, a flag-tag sequence was inserted 5’ to the DGKζ coding region. Overexpression of miR-34a caused a dramatic decrease of DGKζ-WT but not of DGKζ-MM (Figure 2D). Moreover, the levels of flag-SLP76 as a transfection control were not obviously affected by miR-34a. These data demonstrate that miR-34a may directly target DGKζ via seed matches on both 3' UTR and CR. 

### miR-34a enhances T cell activation following TCR cross-linking

**Figure 3:**
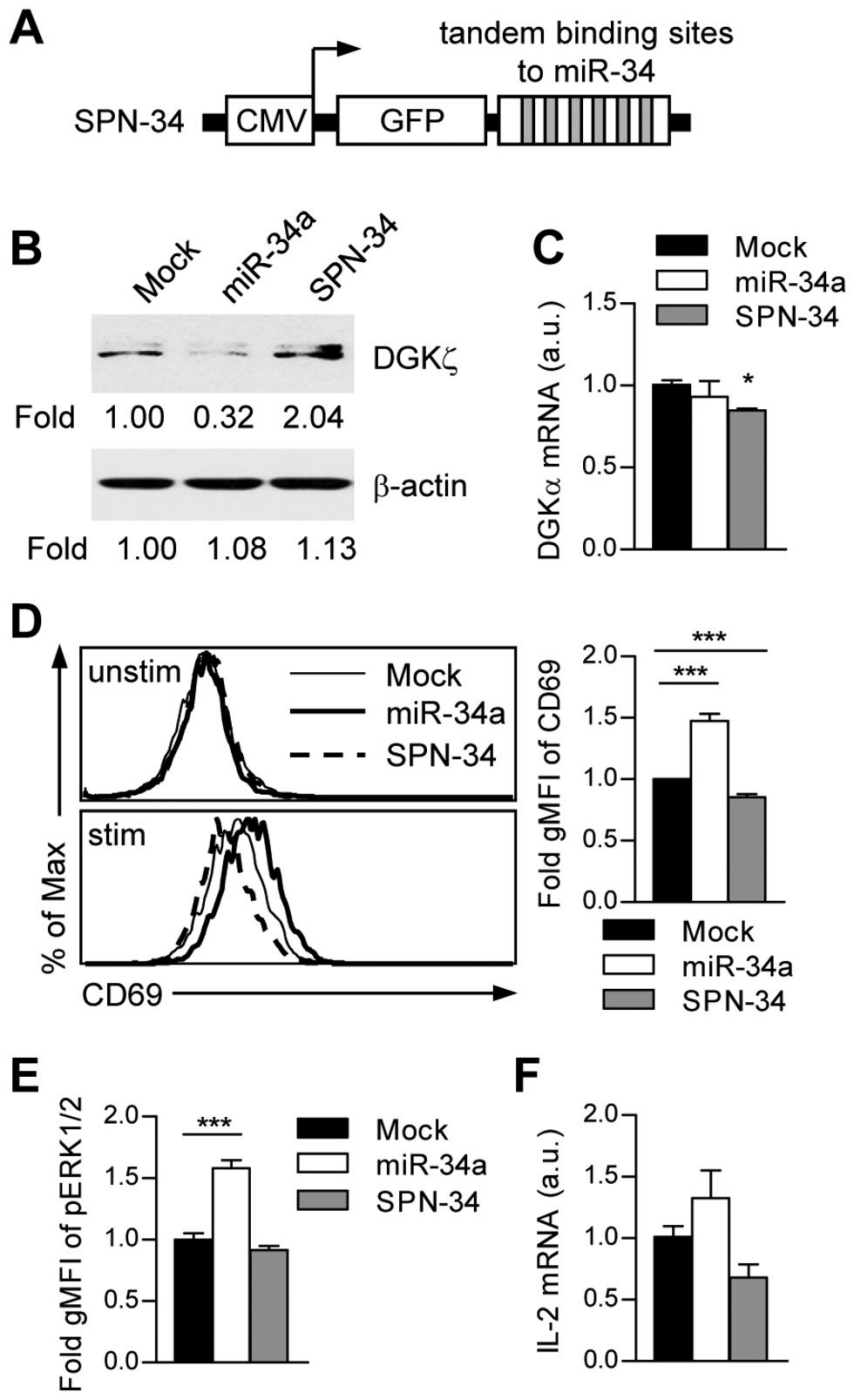
miR-34a enhances T cell activation through repressing DGKζ expression. (**A**) Schematic representation of CMV promoter-driven miR-34 sponge (SPN-34). (**B**) Stable Jurkat T cells transduced with miR-34a or SPN-34 were lysed, and DGKζ protein levels were analyzed by a western blot and quantified with a densitometer. (**C**) DGKα mRNA expression were determined by real-time qPCR (a.u., arbitrary unit; *P < 0.05). (**D**) Jurkat T cells transduced with miR-34a or SPN-34 were stimulated with C305 (1:50000) overnight, followed by FACS analysis of CD69 expression. Bar graphs display mean ± SEM from three experiments (gMFI, geometric mean fluorescence intensity; unstim, unstimulated; stim, stimulated; ***P < 0.001). (**E**) Cells were stimulated with C305 (1:10000, 10 min), fixed, and permeablized. Erk1/2 phosphorylation was determined by intracellular staining and FACS analysis. gMFIs were calculated from triplicates of three experiments (mean ± SEM). (**F**) IL-2 mRNA levels in the indicated cells following 8 hr C305 stimulation were analyzed by real-time qPCR.

 DGKζ functions as a negative regulator in primary T cell responses [10-12]. To access the physiological role of miR-34a in T cells, we first examined whether loss of miR-34a can restore endogenous DGKζ expression using a miR-34a sponge bearing six tandem-binding sites for the miR-34a seed (Figure 3A). A stably transduced miR-34a sponge increased cellular DGKζ protein expression in Jurkat T cells, confirming that DGKζ is a natural target of miR-34a (Figure 3B). DGKα mRNA expression are comparable in these cell lines (Figure 3C). Stimulation of Jurkat T cells transduced with miR-34a using an anti-Jurkat TCR antibody (C305) induced increased expression of the T cell activation marker CD69 and increased Erk1/2 phosphorylation, which were decreased in Jurkat T cells transduced with a miR-34a sponge compared to Mock controls (Figures 3D and 3E). Similar trends of TCR-stimulated induction of IL-2 mRNA were also observed (Figure 3F). Together, our data suggest that miR-34a induced during T cell activation can directly and functionally target DGKζ to promote T cell response.

## Discussion

 DGKζ and DGKα are predominant isoforms of DGKs expressed in T cells [5]. Disruption of DAG metabolism by these two kinases shows synergic effects on T cell development in the thymus and TCR-mediated responses [5,11,32]. miRNAs are fundamentally important regulatory molecules involved in diverse biological processes, including immune cell development and function [16,33]. However, it is unknown whether miRNAs can regulate T cell activation by manipulating DAG signaling. Here, we demonstrate that miR-34a is induced while DGKζ is downregulated following TCR-induced T cell activation. Moreover, miR-34a directly targets DGKζ via its seed matches in both CR and 3' UTR of DGKζ, resulting in enhanced T cell expression of the activation marker CD69.

 Both DGKα and ζ are involved in DAG metabolism. Altered DAG metabolism due to a deficiency of DGKζ or DGKα enhances T cell activation but impairs the induction of anergy in vivo [13]. DGKα mRNA is dependent on FoxO and early growth response gene 2 (Egr2). Sequestration of FoxO in the cytoplasm decreased DGKα transcription following T cell activation, while increasing Egr2 expression in anergic T cells promoted DGKα upregulation [34,35]. Our finding that miR-34a negatively controls DGKζ expression provides mechanistic control of DGKζ expression during T cell activation. Further studies are needed to explore the importance of miR-34a-mediated regulation of DGKζ expression in T cell development and function.

In addition to T cells, DGKζ also regulates the high-affinity receptor for IgE signaling and mast cell activation, controls TLR-induced innate immunity in macrophages and dendritic cells, and modulates the sensitivity to peanut allergens in a murine model [36-38]. miR-34a is widely expressed in immune cells, including dendritic cells, macrophages, mast cells, and B cells for regulation of development, function, or survival [24,39-41]. TLR4 stimulation by lipopolysaccharide in macrophages downregulates miR-34a expression, leading to increased inflammatory response [41]. Upregulation of miR-34a due to genetic ablation of the tuberous sclerosis complex 1 compromises mast cell survival [24]. Future study should determine the role of miR-34a in the control of DGKζ expression in these cell types and the contribution of altered DGKζ expression for miR-34a-mediated cellular processes.
